# Effects of high fat diet-induced obesity on pathophysiology, immune cells, and therapeutic efficacy in systemic lupus erythematosus

**DOI:** 10.1038/s41598-022-21381-3

**Published:** 2022-11-02

**Authors:** Eun Wha Choi, Hee Je Kim, Yun Chan Jung, Hye Sun Go, Je Kyung Seong

**Affiliations:** 1 Department of Veterinary Clinical Pathology, College of Veterinary Medicine and Institute of Veterinary Science, Kangwon National University, 1 Kangwondaehak-gil, Chuncheon-si, Gangwon-do 24341 Republic of Korea; 2 Chaon, 331 Pangyoyeok-ro, Bundang-gu, Seongnam, Republic of Korea; 3 Laboratory of Developmental Biology and Genomics, BK21 Plus Program for Advanced Veterinary Science, Research Institute for Veterinary Science, College of Veterinary Medicine, and Korea Mouse Phenotyping Center, Seoul National University, 599 Gwanak-ro, Gwanak-gu, Seoul, 08826 Republic of Korea; 4 Interdisciplinary Program for Bioinformatics, Seoul National University, 599 Gwanak-ro, Gwanak-gu, Seoul, 08826 Republic of Korea

**Keywords:** Obesity, Systemic lupus erythematosus

## Abstract

Prior studies have suggested a strong link between obesity and autoimmune diseases. This study aimed to evaluate the effects of high fat diet (HFD)-induced obesity on the disease pathogenesis, immune cell infiltration, and therapeutic efficacy in systemic lupus erythematosus (SLE). Treatment with methylprednisolone significantly increased the survival in the control diet group, but not in the HFD group. An HFD significantly increased the incidence of severe proteinuria and glucose intolerance. Regardless of the diet, treatment with methylprednisolone significantly decreased the serum levels of anti-dsDNA antibodies, IL-2, IL-10, and interferon γ-induced protein 10 (IP-10), and improved the renal pathology scores. Treatment with methylprednisolone significantly lowered the serum levels of IL-6, MCP-1, and TNF-α in the control diet group, but not in the HFD group. HFD significantly increased the proportions of CD45^+^ and M1 cells and significantly decreased the proportion of M2 cells in white adipose tissue; methylprednisolone treatment significantly rescued this effect. In the HFD group, methylprednisolone treatment significantly decreased the M1:M2 and increased the Foxp3^+^:RORγt^+^ cell in the spleen compared with the untreated group. These data improve our understanding of the effect of HFD on the therapeutic efficacy of corticosteroids in SLE treatment, which could have clinical implications.

## Introduction

Autoimmune diseases develop when the immune system attacks the body tissues owing to an inappropriate immune response to autoantigens. Approximately 6% of the total population is affected by autoimmune diseases^1^. The underlying causes of autoimmune diseases remain poorly understood, although genetic factors, immunological abnormalities, hormonal abnormalities, and environmental influences are thought to be involved^2^. Recently, epigenetic factors have been reported to contribute to the development of autoimmune diseases^3,4^. Genetic factors have been clearly shown to affect the development of inflammatory autoimmune diseases; however, the agreement rate among identical twins for most diseases is relatively low, suggesting the environmental factors to also be important disease triggers^5^.

The high prevalence of autoimmune diseases correlates with socioeconomic improvement and westernization^6,7^. Obesity and westernized dietary habits (excess of salt, fat, and quantity) are risk factors for metabolic and cardiovascular diseases, while a high body mass index (BMI) is a risk factor for autoimmune diseases^8^. A retrospective study found that a high BMI and obesity prior to adulthood increased the risk of developing multiple sclerosis^9^, while few case studies have suggested that obesity and metabolic syndrome are associated with psoriasis and rheumatoid arthritis^10,11^. Therefore, obesity and metabolic syndrome are probably the most important factors that can lead to inflammatory autoimmune diseases^12^. In animal experiments using disease models, consumption of a high-fat diet (HFD) exacerbated inflammatory bowel disease and collagen-induced arthritis^13,14^. As described above, there is significant evidence to suggest a link between obesity and autoimmune diseases^15^.

Systemic lupus erythematosus (SLE) is a representative systemic autoimmune disease, which is responsible for the formation of autoantibodies. Patients with SLE show a wide range of clinical symptoms, affecting a variety of organs, including the skin, kidneys, nervous system, musculoskeletal system, cardiovascular tissue, and lungs. In a previous study on the relationship between SLE disease activity and obesity, an increased BMI was significantly associated with a higher SLE disease activity index (SLEDAI)^16^. In another study, obesity was not correlated with SLE disease activity, but appeared to be linked to lupus nephritis, cardiovascular morbidities, and a low quality of life^17^.

A recent cohort study showed a significant (85%) increased risk of SLE in obese women compared to those with a normal BMI^18^. Furthermore, another study showed that the obesity rates were higher in SLE patients compared to the general population, and that an HFD could induce dysbiosis^19^. It has further been hypothesized that adipokines and dysbiosis may influence the development of SLE^19^. Other studies have shown that chronic inflammatory conditions tend to worsen in obese SLE patients due to increased oxidative stress, concurrent with an increased expression of C-reactive protein, protein oxidation, and lipid hydrogen peroxide^20^. In a recent cross-sectional study of female SLE patients, patients with an excess body weight showed a higher clinical activity than those with a normal body weight^21^.

Corticosteroids are one of the most commonly used drug classes for the treatment of SLE; these drugs have immunosuppressive and anti-inflammatory effects, triggering an increase in the production of Th2 cytokines and a decrease in the production of Th1 by inhibiting IL-12 secretion from monocytes^22,23^. Monocytes treated with glucocorticoids elevate the secretion of IL-10 and TGF-β and induce the expression of the anti-inflammatory membrane markers CD206, CD163, and CD169^24–26^. Corticosteroid administration is reported to induce dyslipidemia^27^. However, there have been no specific studies on the effect of HFD-induced obesity on SLE treatment comprising corticosteroids.

The NZB/W F1 mouse model is an established SLE animal model. The purpose of this study was to evaluate the effects of HFD on disease pathogenesis, immune cells, and therapeutic efficacy in SLE using the NZB/W F1 mouse model.

## Results

### Mouse groups

Mice were divided into four groups as follows: CN, chow diet and non-treatment control (saline, 200 μl/day); CP, chow diet and methylprednisolone treatment (5 mg/kg/day); HN, high-fat diet and non-treatment control (saline, 200 μl/day); and HP, high-fat diet and methylprednisolone treatment (5 mg/kg/day).

### Survival rates

**Figure 1:**
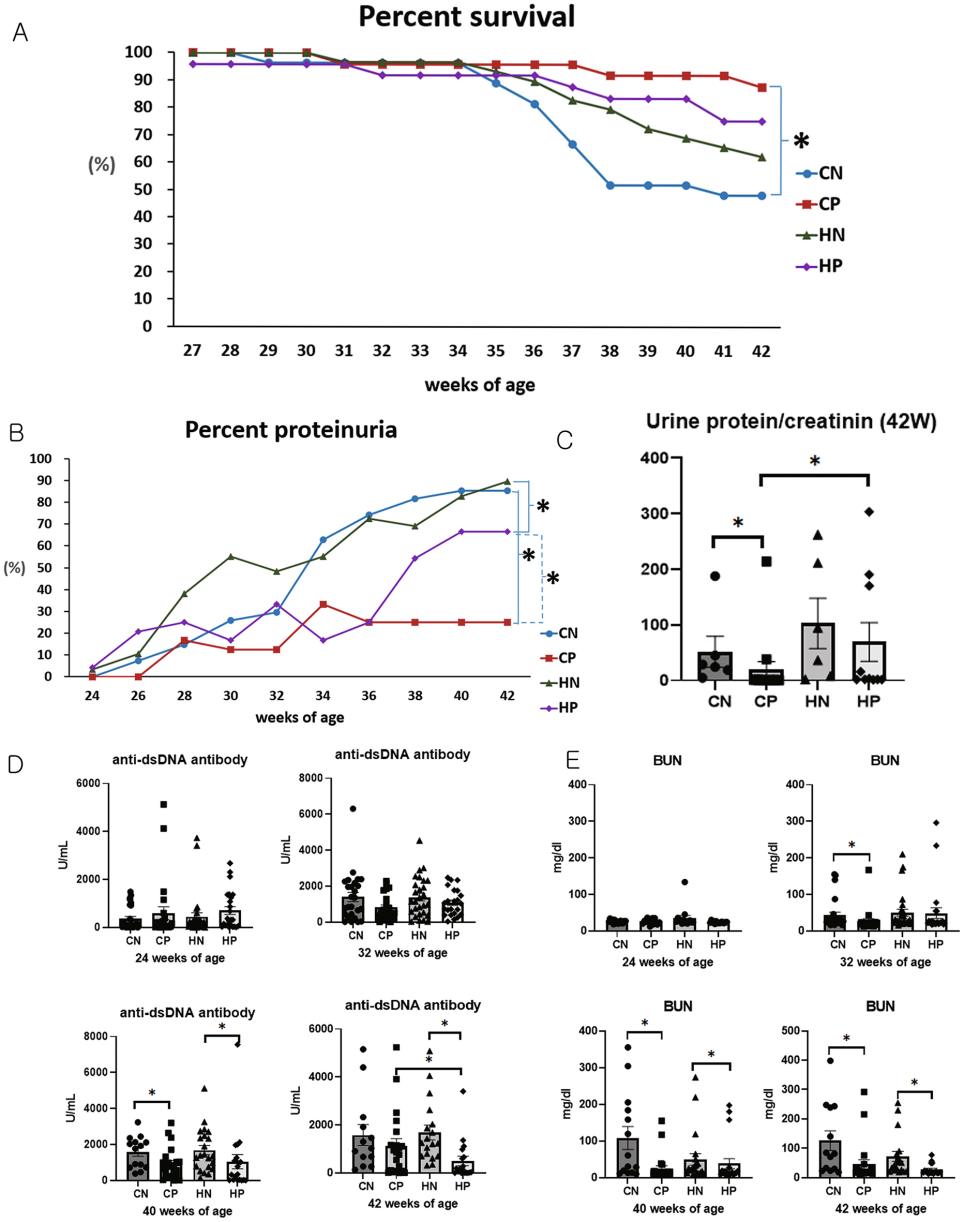
Survival, progression of severe proteinuria, and serology. (**A**) Survival, (**B**) incidence of severe proteinuria (≥ 300 mg/dl), (**C**) urine protein to creatinine ratio at 42 weeks of age, (**D**) serum levels of anti-dsDNA antibodies, (**E**) blood urea nitrogen concentration. Data from (**A**) and (**B**) were analyzed using Kaplan–Meier curves and the log-rank test, and data from (**C**–**E**) were compared using the Kruskal–Wallis test followed by Mann–Whitney *U* test. *Significant differences between groups (*P* < 0.05) are marked with an asterisk. *CN* chow diet and non-treatment control (saline, 200 μl/day), *CP* chow diet and methylprednisolone treatment (5 mg/kg/day), *HN* high fat-diet and non-treatment control (saline, 200 μl/day), *HP* high fat-diet and methylprednisolone treatment (5 mg/kg/day).

The groups showed a significant difference in survival (log-rank test, *P* = 0.015). The survival rate was significantly higher in the CP group than in the CN group (pairwise, *P* = 0.003); however, there was no significant difference in survival between the HN and HP groups (Fig. 1A). In other words, methylprednisolone treatment significantly increased the survival rate under the control diet, but not under an HFD.

### Incidence of severe proteinuria

We observed a significant difference in the incidence of severe proteinuria between the groups (log-rank test, *P* < 0.001). Severe proteinuria was significantly lower in the CP group than in the CN group (pairwise, *P* < 0.001), and was also lower in the HP group than in the HN group (pairwise, *P* = 0.015). The incidence of severe proteinuria was also significantly lower in the CP group than in the HP group (pairwise, *P* = 0.01; Fig. 1B). In other words, the incidence of severe proteinuria was significantly lowered by methylprednisolone treatment, while HFD significantly increased the incidence, even in the methylprednisolone treatment group.

At 42 weeks, the urinary protein to creatinine ratio (UP/C) showed significant differences between the groups (Kruskal–Wallis, *P* = 0.001); UP/C was significantly lower in the CP group compared to the CN group (Mann–Whitney *U*, *P* = 0.006), and in the CP group compared to the HP group (Mann–Whitney *U*, *P* = 0.001). However, there was no significant difference in the UP/C between the HN and HP groups (Fig. 1C).

### Anti-dsDNA antibody concentrations, BUN and UP/C

Serum anti-dsDNA antibody concentrations obtained at 24 and 32 weeks of age showed no significant difference among the groups (Kruskal–Wallis, *P* = 0.244 and *P* = 0.190, respectively); however, significant differences were observed at 40 weeks of age (Kruskal–Wallis, *P* = 0.007). At this point, serum anti-dsDNA antibody concentrations in the CP group were significantly lower than those in the CN group (Mann–Whitney *U*, *P* = 0.038), while the serum anti-dsDNA antibody concentrations in the HP group were significantly lower than those in the HN group (Mann–Whitney *U*, *P* = 0.007). Concentrations at 42 weeks of age differed significantly among the groups (Kruskal–Wallis, *P* = 0.001); concentrations in the HP group were significantly lower than those in the HN group (Mann–Whitney *U*, *P* < 0.001), and those in the HP group were significantly lower than those in the CP group (Mann–Whitney *U*, *P* = 0.043, Fig. 1D).

Blood urea nitrogen levels showed no significant difference at 24 weeks of age (Kruskal–Wallis, *P* = 0.236); however, those obtained at 32, 40, and 42 weeks of age were significantly different among the groups (Kruskal–Wallis, *P* < 0.001, *P* = 0.001, and *P* < 0.001, respectively). BUN levels at 32, 40, and 42 weeks of age were significantly lower in the CP group than in the CN group (Mann–Whitney *U*, *P* < 0.001, *P* = 0.001, and *P* = 0.001, respectively), and those in the HP group were significantly lower than those in the HN group at 40 and 42 weeks of age (Mann–Whitney *U*, *P* = 0.027 and *P* = 0.001, respectively Fig. 1E).

### Intraperitoneal glucose tolerance test (IPGTT)

The glucose levels were significantly different among the groups at 0, 15, 30, 60, 90, and 120 min in the IPGTT (Kruskal–Wallis, *P* = 0.013, *P* < 0.001, *P* = 0.004, *P* = 0.01, *P* = 0.046, and *P* < 0.001, respectively). The CP group recovered to below the baseline at 120 min after the IPGTT. When fed a chow diet, the CP group showed significantly lower blood glucose levels at 15, 30, and 120 min than the control group (CN) (Mann–Whitney *U*, *P* = 0.007, *P* = 0.002, and *P* = 0.006, respectively). However, when fed the HFD, there was no significant difference in blood glucose levels at each time point between the treatment (HP) and control groups (HN).

**Figure 2:**
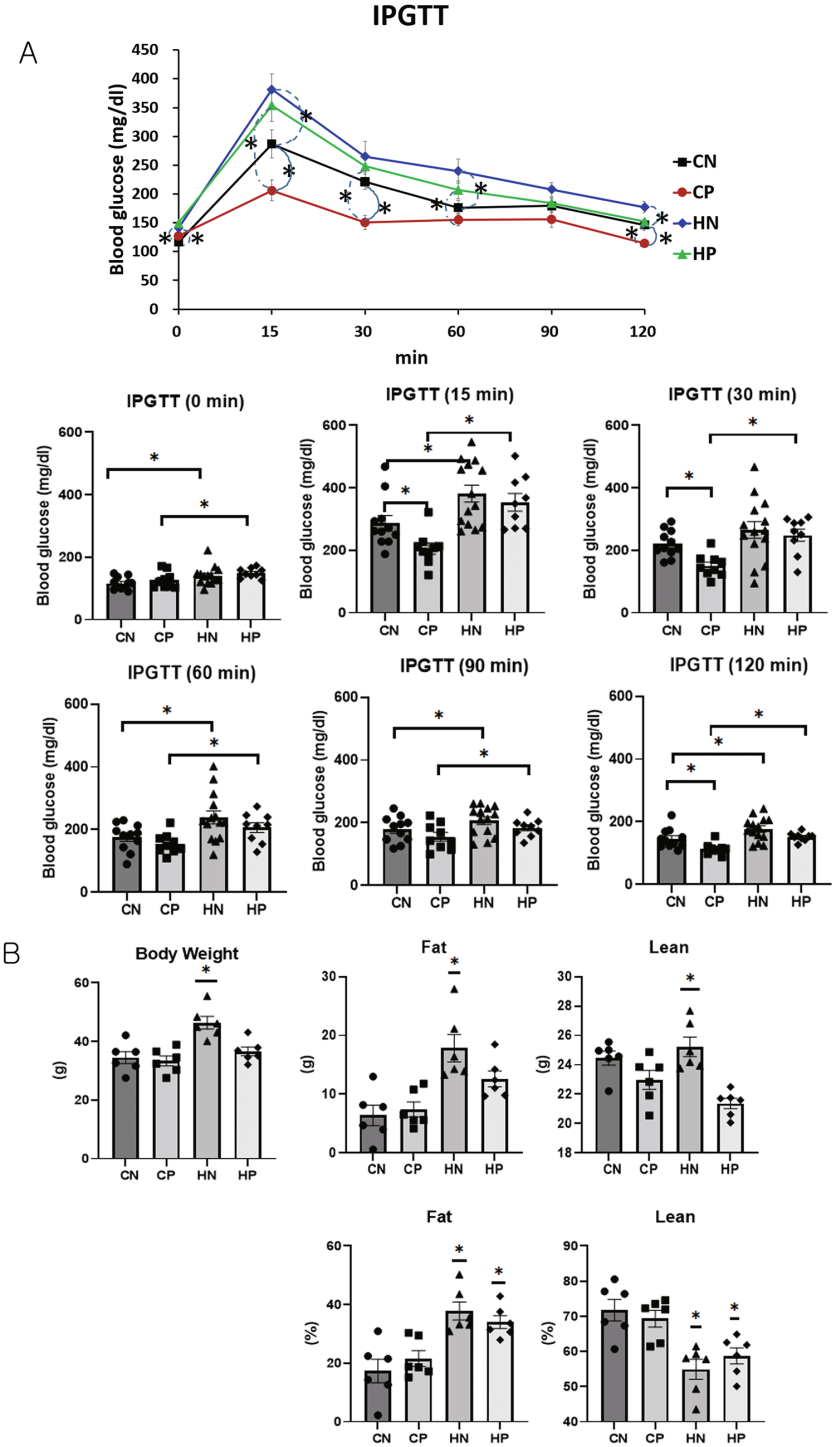
Intraperitoneal glucose tolerance test and body composition. (**A**) Intraperitoneal glucose tolerance test at 36 weeks of age (all mice in the first experiment, CN: n = 11, CP: n = 9, HN: n = 14, HP: n = 9). (**B**) Body weight (total mass, g), total weight of fat (g), lean mass (g), percentage of fat, and percentage of lean mass measured by dual-energy X-ray absorptiometry at 42 weeks of age (n = 6 per group). The data from (**A**) were compared using the Kruskal–Wallis test followed by the Mann–Whitney *U* test. The data from (**B**) were compared among groups using one-way analysis of variance (ANOVA) followed by post hoc Tukey's multiple-comparison tests (n = 6 per group). A *P*-value < 0.05 was considered statistically significant. *CN* chow diet and non-treatment control (saline, 200 μl/day), *CP* chow diet and methylprednisolone treatment (5 mg/kg/day), *HN* high fat-diet and non-treatment control (saline, 200 μl/day), *HP* high fat-diet and methylprednisolone treatment (5 mg/kg/day).

In the untreated group, the control diet group (CN) had significantly lower glucose levels at 0, 15, 60, and 120 min than the HFD group (HN) (Mann–Whitney *U* test, *P* = 0.029, *P* = 0.009, *P* = 0.038, and *P* = 0.044, respectively). In the methylprednisolone treatment condition, the control diet group (CP) had significantly lower blood glucose levels at 0, 15, 30, 60, and 120 min than the HFD group (HP) (Mann–Whitney *U*, *P* < 0.001, *P* = 0.002, *P* = 0.04, *P* = 0.038, and *P* < 0.001, respectively, Fig. 2A). Therefore, HFD appears to affect the glucose tolerance.

### Body composition

The HN group showed a significantly higher body weight and lean weight (g) than the other groups (CN, CP, and HP groups) (analysis of variance [ANOVA] and Tukey, *P* < 0.001). Additionally, the HN group had a significantly higher fat weight (g) than the CN and CP groups (ANOVA and Tukey, *P* < 0.001). The HN and HP groups showed significantly higher fat proportions (%) than the CN and CP groups (ANOVA and Tukey, *P* < 0.001), and the lean proportion (%) of the HN and HP groups was significantly lower than that of the CN and CP groups (ANOVA and Tukey, *P* < 0.001; Fig. 2B).

### Indirect calorimetry

Body weight was significantly higher in the HN group than in the other groups (ANOVA and Tukey, *P* < 0.001, Supplementary Fig. 1A), with no statistically significant difference in the activity between the groups (Supplementary Fig. 1B). The CP group showed higher ingestion of FEED1 (g/kg/h) than the HN group (ANOVA and Tukey, *P* = 0.007). FEED (kcal/kg/h) was further ingested by the CP group more than the CN and HN groups (ANOVA and Tukey, *P* = 0.004; Supplementary Fig. 1C). EE (HEAT, kcal/kg/h) was also significantly higher in the CP group than in the HN group (ANOVA and Tukey, *P* = 0.034, Supplementary Fig. 1D), and RER was significantly higher in the CN and CP groups than in the HN and HP groups (ANOVA and Tukey, *P* < 0.001, Supplementary Fig. 1E). VO_2_ and VCO_2_ were significantly higher in the CP group than in the HN group (ANOVA and Tukey test, *P* = 0.038 and *P* = 0.005, Supplementary Fig. 1F,G).

### Flow cytometric determination of the T cell, macrophage, and T helper subset proportions in the spleen

**Figure 3:**
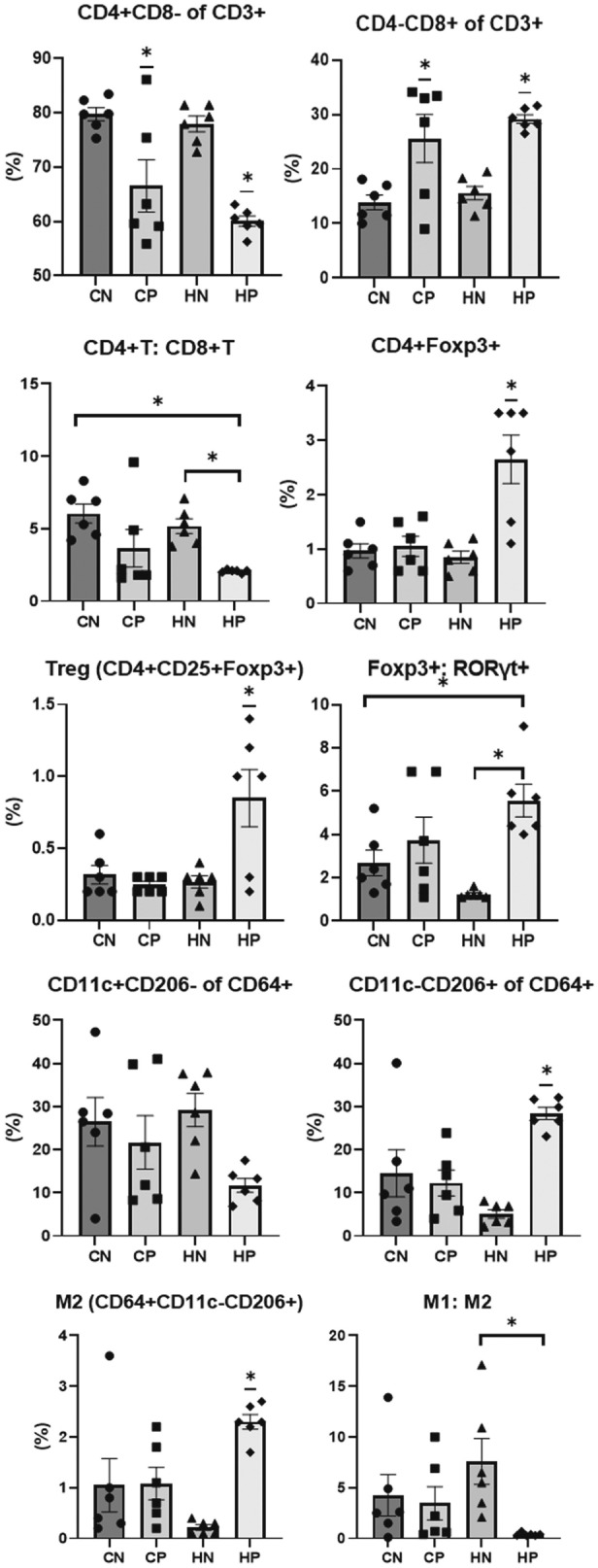
Flow cytometric determination of T cell, T helper cell subset, and macrophage proportions in the spleen. Proportions of CD4^+^CD8^−^ of CD3^+^ cells and CD4^−^CD8^+^ of CD3^+^ cells, and the ratio of CD4^+^CD8^−^/CD4^−^CD8^+^ cells. CD4^+^Foxp3^+^ cells, Treg cells (CD4^+^CD25^+^ Foxp3^+^), Foxp3^+^:RORγt^+^, CD11c^+^CD206^−^ of CD64^+^ cells, CD11c^−^CD206^+^ of CD64^+^ cells, M2 (CD64^+^CD11c^−^CD206^+^), and M1:M2 in spleen (n = 6 per group). Data obtained from each group were compared using one-way analysis of variance (ANOVA) followed by post hoc Tukey's multiple comparison tests. *Significant (P < 0.05) difference. *CN* chow diet and non-treatment control (saline, 200 μl/day), *CP* chow diet and methylprednisolone treatment (5 mg/kg/day), *HN* high fat-diet and non-treatment control (saline, 200 μl/day), *HP* high fat-diet and methylprednisolone treatment (5 mg/kg/day).

The proportion of CD4^+^ cells was significantly lower and proportion of CD8^+^ cells was significantly higher in the CP and HP groups than in the CN and HN groups (ANOVA and Tukey, *P* < 0.001 and *P* < 0.001, respectively). Regardless of diet, the proportion of CD4^+^ cells was significantly lower and proportion of CD8^+^ cells was significantly higher following methylprednisolone treatment. The HP group showed a significantly higher proportion of CD11c^−^CD206^+^ cells than the other groups (CN, CP, and HN) (ANOVA and Tukey, *P* = 0.001). The HP group had a significantly lower M1:M2 ratio and a significantly higher proportion of CD64^+^CD11c^−^CD206^+^ cells (M2) than the HN group (ANOVA and Tukey, *P* = 0.002). The proportions of CD4^+^Foxp3^+^ cells and Treg cells (CD4^+^CD25^+^Foxp3^+^) were significantly higher in the HP group than in the other groups (CN, CP, and HN groups) (ANOVA and Tukey, *P* = 0.001 and *P* = 0.002, respectively), and the Foxp3^+^:ROR-γt^+^ ratio in the HP group was significantly higher than that in the CN and HN groups (ANOVA and Tukey, *P* = 0.003). The flow cytometry data for splenocytes are shown in Fig. 3.

### Immune cell analysis in stromal vascular fraction (SVF) extracted from epididymal white adipose tissue (eWAT)

**Figure 4:**
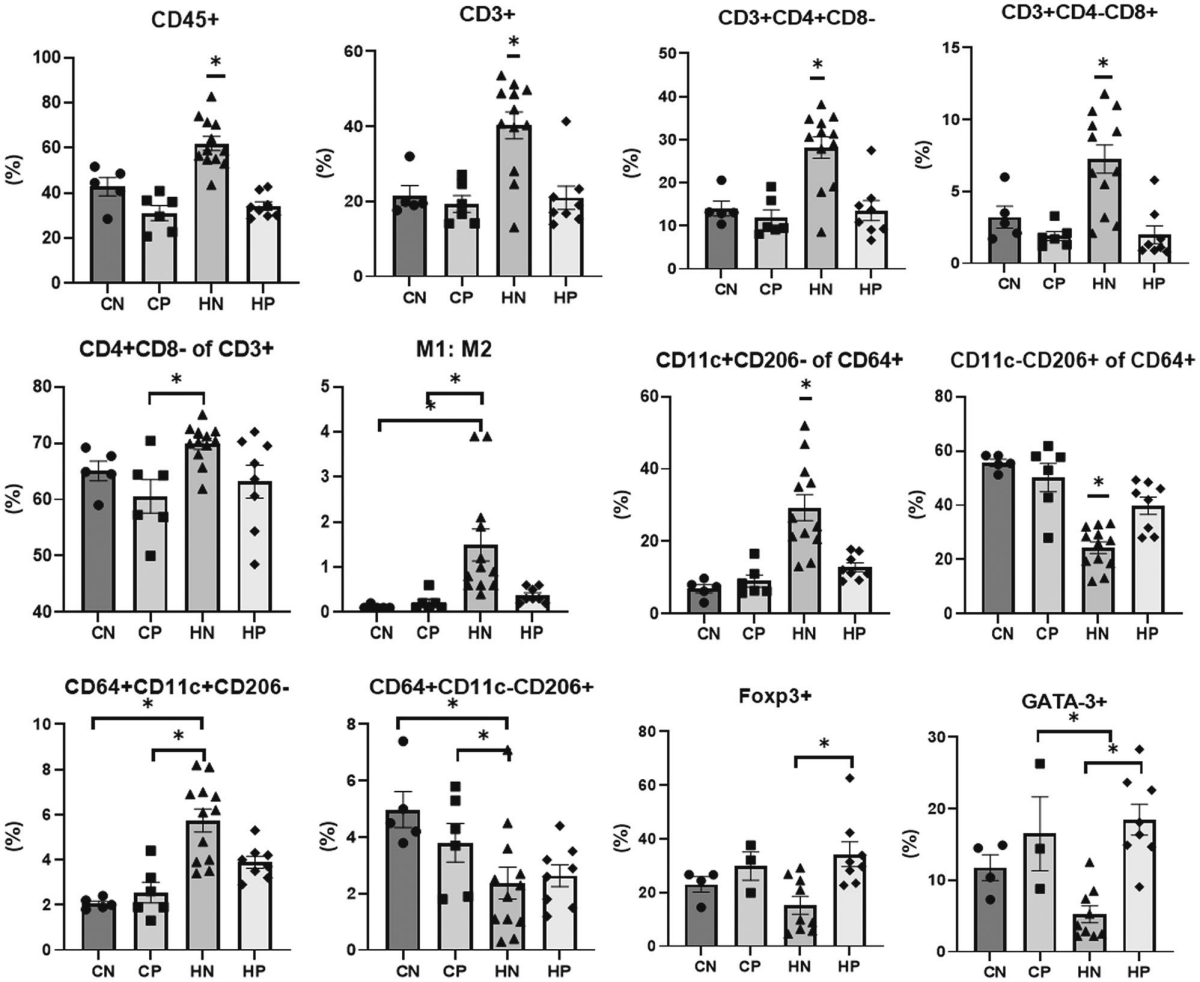
The percentages of inflammatory cells in stromal vascular cells from epididymal white adipose tissue analysed by flow cytometry. The percentages of leukocytes, CD45^+^CD3^+^, CD3^+^CD4^+^CD8^−^, CD3^+^CD4^−^CD8^+^, CD4^+^CD8^−^ of CD3^+^ cells, M1:M2, CD11c^+^CD206^−^ of CD64^+^ cells, CD11c^−^CD206^+^ of CD64^+^ cells, M1 (CD64^+^CD11c^+^CD206^−^), M2 (CD64^+^CD11c^−^CD206^+^), Foxp3^+^ cells, and GATA-3^+^ cells in stroma vascular cells from eWAT (n = 5–12 per group). Data obtained from each group were compared using one-way analysis of variance (ANOVA) followed by post hoc Tukey's multiple comparison tests. *Significant (*P* < 0.05) difference. *eWAT* epididymal white adipose tissue, *SVF* stromal vascular cells, *CN* chow diet and non-treatment control (saline, 200 μl/day), *CP* chow diet and methylprednisolone treatment (5 mg/kg/day), *HN* high fat-diet and non-treatment control (saline, 200 μl/day), *HP* high fat-diet and methylprednisolone treatment (5 mg/kg/day).

In the flow cytometric analysis of SVF extracted from eWAT, the proportion of CD45^+^ cells, CD3^+^ cells, CD3^+^CD4^+^ cells, and CD3^+^CD8^+^ cells in the HN group was significantly higher than that in other groups (CN, CP, and HP) (all parameters: ANOVA and Tukey, *P* < 0.001). The proportion of CD11c^+^CD206^−^ cells was significantly higher and the proportion of CD11c^−^CD206^+^ cells was significantly lower in the HN group compared to the other groups (CN, CP, and HP) (all parameters: ANOVA and Tukey, *P* < 0.001). The HN group had a significantly higher M1:M2 ratio than either the CN or CP groups (ANOVA and Tukey test, *P* = 0.003). Furthermore, the HP group showed a significantly higher proportion of Foxp3^+^ and GATA3^+^ cells than the HN group (ANOVA and Tukey, *P* = 0.011 and *P* < 0.001, respectively). The flow cytometry data of SVF extracted from fat are shown in Fig. 4.

### Histopathological lesions of the kidneys

**Figure 5:**
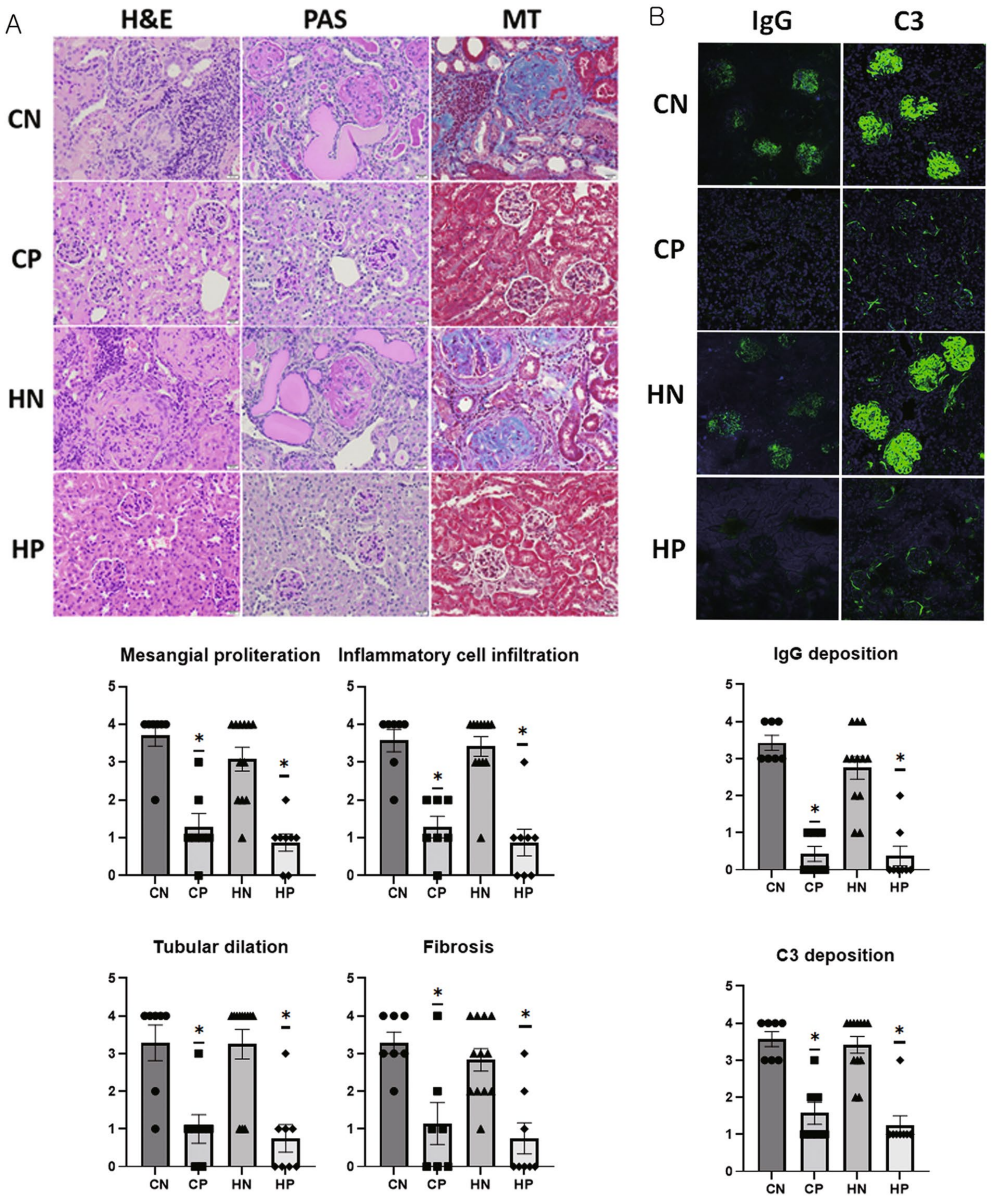
Histopathological and immunofluorescent analysis of kidneys. (**A**) Hematoxylin and eosin (H&E), periodic acid-Schiff (PAS) reagent, and Masson’s trichrome staining of kidneys obtained from mice at the end of the experiment (original magnification: × 400). Inflammatory cell infiltration and mesangial proliferation were scored on a graded scale from 0 (none) to 4 (severe). (**B**) IgG and C3 deposition in kidneys (original magnification: × 200). Fluorescence staining intensities of IgG and C3 deposits were graded as 0 (none), 1+ (mild), 2+ (moderate), 3+ (moderate to strong), or 4+ (strong). Intergroup analysis was performed using ANOVA followed by Tukey’s multiple comparison post-hoc tests. *Significant (*P* < 0.05) difference. *CN* chow diet and non-treatment control (saline, 200 μl/day, n = 7), *CP* chow diet and methylprednisolone treatment (5 mg/kg/day, n = 7), *HN* high fat-diet and non-treatment control (saline, 200 μl/day, n = 12), *HP* high fat-diet and methylprednisolone treatment (5 mg/kg/day, n = 8).

Large numbers of inflammatory cell infiltrations, mesangial proliferation, dilated tubules, and fibrosis were observed in the CN and HN groups on histopathological evaluation of the kidneys. However, the degree of inflammatory cell infiltration, mesangial proliferation, tubular dilation, and fibrosis was significantly lower in the CP and HP groups (all parameters: ANOVA and Tukey, *P* < 0.001, Fig. 5A).

### IgG and C3 infiltration in the kidneys

The degrees of IgG and C3 infiltration in glomeruli were found to be significantly lower in the CP and HP groups than in the CN and HN groups (ANOVA and Tukey, IgG: *P* < 0.001 and C3: *P* < 0.001, Fig. 5B).

### Serum cytokines

Serum levels of IFN-γ, IL-2, IL-10, IP-10, IL-6, and TNF-α were significantly different among all the groups (Kruskal–Wallis, *P* = 0.016, *P* = 0.005, *P* = 0.001, *P* < 0.001, *P* = 0.048, and *P* = 0.001, respectively). Serum levels of IL-2, IL-10, and IP-10 were significantly lower in the CP group than in the CN group (Mann–Whitney *U*, *P* = 0.035, *P* = 0.009, *P* < 0.001, respectively), and were also significantly lower in the HP group than in the HN group (Mann–Whitney *U*, *P* = 0.008, *P* = 0.001, *P* = 0.001, respectively).

**Figure 6:**
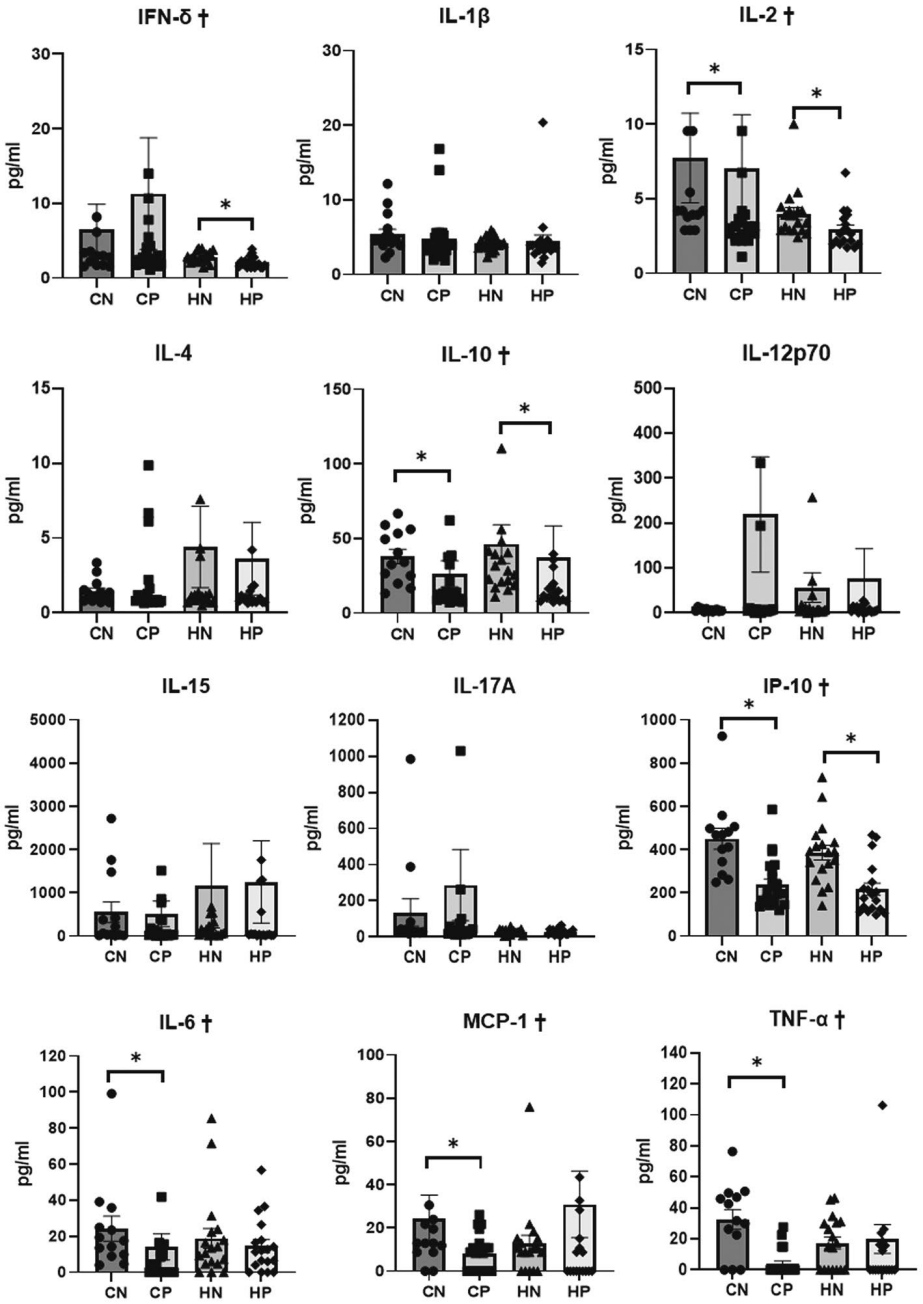
Serum levels of various cytokines. Data are expressed as the mean ± SEM. Data obtained from each group were compared using the Kruskal–Wallis test (^†^*P* < 0.05), followed by the Mann–Whitney *U* test (*). *Significant (*P* < 0.05) differences between the CN and CP groups or between the HN and HP groups are marked with an asterisk. *CN* chow diet and non-treatment control (saline, 200 μl/day, n = 13), *CP* chow diet and methylprednisolone treatment (5 mg/kg/day, n = 21), *HN* high fat-diet and non-treatment control (saline, 200 μl/day, n = 18), *HP* high fat-diet and methylprednisolone treatment (5 mg/kg/day, n = 18).

Serum levels of IL-6, MCP-1 (CCL2), and TNF-α were significantly lower in the CP group than in the CN group (Mann–Whitney *U*, *P* = 0.007, *P* = 0.039, and *P* < 0.001, respectively); however, no significant difference was found between the HN and HP groups. In other words, serum levels of IL-6, MCP-1, and TNF-α were not significantly lowered by the methylprednisolone treatment in the HFD group (Fig. 6).

### Serum adipokines and insulin

**Figure 7:**
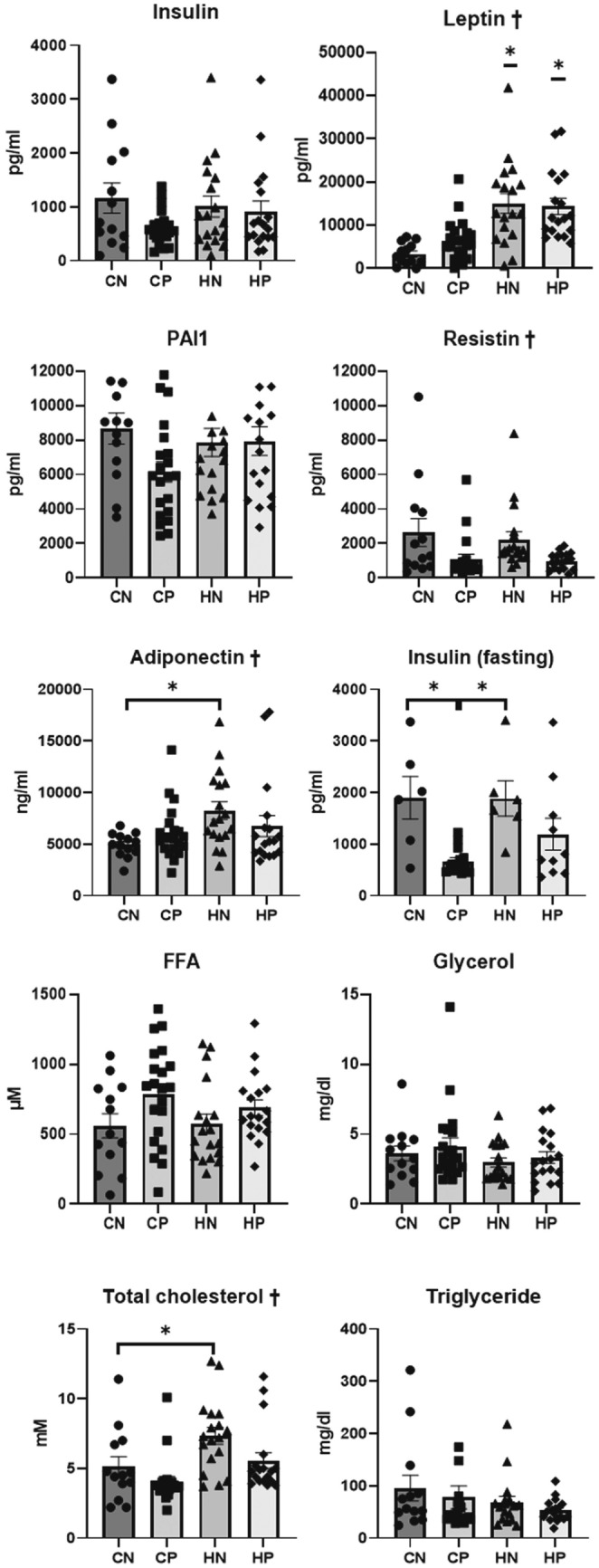
Serum levels of free fatty acids, glycerol, total cholesterol, triglycerides, insulin, and adipokines. Data are expressed as mean ± SEM. Data obtained from each group were compared using one-way analysis of variance (ANOVA, ^†^*P* < 0.05) followed by post hoc Tukey's multiple comparison tests. (*P < 0.05). *CN* chow diet and non-treatment control (saline, 200 μl/day, n = 13), *CP* chow diet and methylprednisolone treatment (5 mg/kg/day, n = 21), *HN* high fat-diet and non-treatment control (saline, 200 μl/day, n = 18), *HP* high fat-diet and methylprednisolone treatment (5 mg/kg/day, n = 18).

Regardless of the treatment, the serum levels of leptin were significantly higher in the HFD groups (HN and HP groups) than in the control diet groups (CN and CP groups) (ANOVA and Tukey, *P* < 0.001). The serum resistin levels were significantly different among all the groups (ANOVA, *P* = 0.016), while there was no significant difference in the serum insulin levels between the groups without fasting. The serum insulin levels during overnight fasting were also significantly lower in the CP group than in the CN group (ANOVA and Tukey, *P* = 0.003); however, there was no significant difference in these levels between the HN and HP groups. Serum adiponectin levels were significantly higher in the HN group than in the CN group (ANOVA and Tukey, *P* = 0.042, Fig. 7).

### Serum levels of free fatty acids, glycerol, total cholesterol, and triglycerides

Serum levels of free fatty acids, glycerol, and triglycerides obtained at 42 weeks of age showed no significant difference between the groups. Serum levels of total cholesterol obtained at 42 weeks of age in the CN and CP groups were significantly lower than those in the HN group (ANOVA and Tukey, *P* < 0.001, Fig. 7).

### Cytokine and chemokine levels in kidney extracts

**Figure 8:**
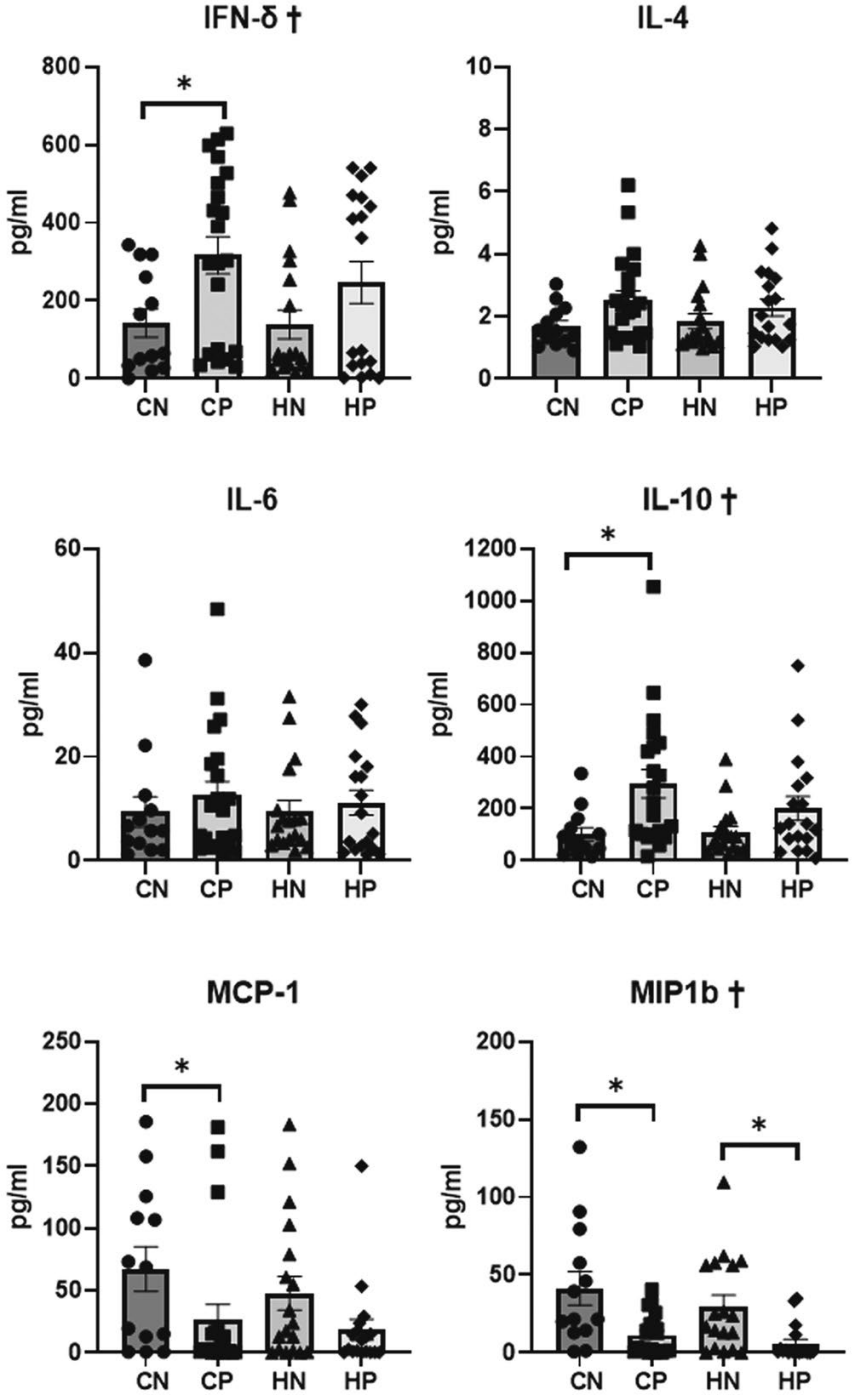
Cytokines and chemokine levels in kidney extracts. Data are expressed as mean ± SEM. Data obtained from each group were compared using the Kruskal–Wallis test (^†^*P* < 0.05), followed by the Mann–Whitney *U* test (*). *Significant (*P* < 0.05) differences between the CN and CP groups or between the HN and HP groups are marked by an asterisk. *CN* chow diet and non-treatment control (saline, 200 μl/day, n = 13), *CP* chow diet and methylprednisolone treatment (5 mg/kg/day, n = 21), *HN* high fat-diet and non-treatment control (saline, 200 μl/day, n = 18), *HP* high fat-diet and methylprednisolone treatment (5 mg/kg/day, n = 18).

The IL-10, IFN-γ, and MIP-1b (CCL4) levels in kidney extracts were significantly different among the groups (Kruskal–Wallis, *P* = 0.007, *P* = 0.034, and *P* = 0.001, respectively; Fig. 8). The MIP-1b levels in kidney extracts were significantly lower in the CP group than in the CN group (Mann–Whitney *U*, *P* = 0.008), and significantly lower in the HP group than in the HN group (Mann–Whitney *U*, *P* = 0.001). The IFN-γ and IL-10 levels in kidney extracts were significantly higher and MCP-1 levels in kidney extracts were significantly lower in the CP group than in the CN group (Mann–Whitney *U*, *P* = 0.005 and *P* = 0.032, respectively); however, there was no significant difference in these levels between the HN and HP groups.

## Discussion

It is difficult to determine the long-term effect of HFD on disease exacerbation or treatment efficacy in human studies because of the long lifespan and variations in numerous other environmental factors in humans. Studies using animal models can be performed in a controlled environment and are very helpful in the study of disease symptoms and treatment effects of human diseases. In this study, we investigated the effect of HFD-induced obesity on the treatment efficacy of methylprednisolone over a long period (longer than the average lifespan) in a mouse SLE disease model. Our results strongly indicate that the treatment effect of methylprednisolone was greatly affected by diet.

Overall, this study revealed that treatment with methylprednisolone significantly increased the survival rates in the control diet group, but not in the HFD group. An HFD significantly increased the incidence of severe proteinuria, and increased the serum glucose levels as observed following the glucose tolerance test. Regardless of the type of diet, treatment with methylprednisolone significantly decreased the serum levels of anti-dsDNA antibody and BUN and lowered the serum levels of IL-2, IL-10, and IP-10.

Serum levels of IL-2 were significantly higher in SLE patients than in healthy controls^28^, which may be explained by the chronic activation of T lymphocytes in SLE patients^29^. According to a previous study, serum levels of IL-10 were significantly increased in patients with SLE, and the serum levels of IL-10 correlated with the serum levels of anti-dsDNA antibodies^30^. IP-10 (chemokine ligand 10, CXCL10) is an IFN-regulated chemokine, which strongly correlates with SLE disease activity^31^. The serum IP-10 levels of active SLE patients were significantly higher than those of healthy controls and inactive SLE patients^32^. Furthermore, serum IP-10 levels were positively correlated with the SLE disease activity, including the SLEDAI^32–36^. IP-10 promotes the migration of macrophages, dendritic cells, NK cells, and activated T lymphocytes, together with their receptor (CXC receptor 3), to inflammatory sites (CXCR3)^36,37^.

Treatment with methylprednisolone significantly lowered the serum levels of IL-6, MCP-1 (CCL2), and TNF-α in the control diet group, but not in the HFD group. Previous studies have shown that the serum levels of IL-6, MCP-1 (CCL2), and TNF-α are elevated in SLE patients; serum levels of MCP-1 were also significantly higher in SLE patients than in healthy controls, and serum levels of MCP-1 were positively correlated with SLEDAI^38^. Serum levels of TNF-α and IL-6 were significantly elevated in patients with SLE compared with that of healthy controls^29,39,40^. Serum levels of TNF-α were strongly correlated with the serological parameters of disease activity, such as serum levels of anti-dsDNA antibody, C-reactive protein levels, and erythrocyte sedimentation rate, and were also correlated with the clinical parameters of disease activity, such as the European Consensus Lupus Activity Measurement (ECLAM) score and anemia^40^. Injection of recombinant human IL-6 into young female NZB/W F1 mice (6 months of age) accelerated membranoproliferative glomerulonephritis by upregulating the expressions of mesangial MHC class II and glomerular intercellular adhesion molecule-1 (ICAM-1)^41^. In contrast, chronic treatment with anti-IL-6 monoclonal antibodies or anti-IL-6 receptor antibodies from 3 months of age significantly decreased the mortality, incidence of proteinuria, and serum levels of anti-dsDNA antibody^42,43^.

In the white adipose tissue of obese patients, the number of M1 macrophages, which secrete TNF-α and IL-6, is often elevated, resulting in local and systemic low-grade inflammation and insulin resistance^44^. HFD-induced obesity elevates the expression of numerous inflammatory genes, including MCP-1, in the adipose tissue and blood^45,46^. An elevated expression of MCP-1 in adipose tissue contributes to macrophage infiltration and induces insulin resistance and hepatic steatosis associated with obesity^46^. Contrary to expectations, compared to a control diet, an HFD did not significantly increase the serum levels of IL-6, MCP-1, or TNF-α in old NZB/W F1 mice (advanced stage of SLE). However, methylprednisolone treatment of HFD mice did not significantly reduce their serum levels of IL-6, MCP-1, TNF-α, or kidney levels of MCP-1. In other words, HFD nullified the effects of methylprednisolone on these cytokines and SLE symptoms (i.e., increased survival or decreased proteinuria).

In this study, serum leptin levels were found to be significantly higher in the HFD group than in the control diet group. According to previous studies, leptin increases the survival of autoreactive T cells and reduces the number of Treg cells in mouse models of SLE^47,48^. Furthermore, deficient leptin signaling reduces the degree of SLE lesions in MRL/lpr mice, while leptin increases the Th17 responses in NZB/W F1 mice and normal human CD4+ T cells by inducing the RORγt expression^49,50^.

In a recent study on the causal relationship between SLE and HFD and metabolic syndrome, an imbalance of adipokine production in metabolic syndrome was found to exacerbate inflammation, while the metabolic syndrome was observed to predispose SLE patients to the development of chronic kidney disease and diabetes^51^. Dysregulation of cytokines and adipokines is thought to contribute to the complex association among autoimmunity, obesity, inflammation and atherosclerosis^51^.

HFD significantly increased the proportion of CD45+ and M1 cells and significantly decreased the proportion of M2 cells in white adipose tissue, both of which were significantly rescued by methylprednisolone treatment. In the HFD group, the methylprednisolone treatment group (HP) showed a significantly decreased M1:M2 ratio, as well as a significantly increased ratio of the master regulator of Treg–Th17 (Foxp3+:RORγt+) in the spleen compared with the control group (HN). In a previous study, glucocorticoid treatment was found to increase the expression of anti-inflammatory membrane markers, such as CD206 (M2 marker), in monocytes^25^. Regardless of diet, the proportion of CD4+ cells was significantly lower and the proportion of CD8+ cells was significantly higher during methylprednisolone treatment. A previous study reported that the proportion of CD8+ T cells was significantly decreased and the CD4+:CD8+ T cell ratio was significantly increased in the peripheral blood of SLE patients compared with the normal control group^52^.

The results on the survival rate and occurrence of severe proteinuria in this study suggest that obesity induced by HFD significantly reduced the therapeutic effect of methylprednisolone in SLE, in a process probably involving proinflammatory cytokines such as IL-6, MCP-1 and TNF-α, and adipokines.

There are obvious limitations to evaluating the therapeutic effect of SLE in a mouse model. Firstly, SLE in humans is characterized by a variety of clinical symptoms targeting multiple organs, including the skin, joints, and kidneys; conversely, the mouse SLE model used in this experiment was primarily characterized by the occurrence of lupus nephritis^53^. Therefore, although there are various evaluation criteria for clinical symptoms in humans, only the survival rate and incidence of severe proteinuria were evaluated as important clinical indicators in this experiment. Several factors, including the consumption of various micronutrients, exercise, and environmental factors other than diet, may also exert a significant influence on SLE pathology; however, this study only explored the effects of an HFD.

In this study, we evaluated the effects of metabolic environment changes induced by HFD on the disease pathogenesis, number of immune cells, and therapeutic efficacy in a murine model of SLE. The data we obtained here using the NZB/W F1 model mice may present significant translational potential for further understanding the effect of HFD on the therapeutic efficacy of corticosteroids, which may help develop better therapeutic strategies based on dietary control.

## Materials and methods

### Experimental animals and groups

Female NZB/W F1 mice aged 4 weeks were purchased from the Jackson Laboratory (Bar Harbor, ME, USA) and were acclimatized 2 weeks prior to the experiment. In the first experiment, 12, 9, 14, and 9 mice were assigned to the CN, CP, HN, and HP groups, respectively, while in the second experiment 15 mice were assigned to each group. The number of experimental animals was determined with reference to the number of animals used in previous papers^4,54,55^. The mice received different diets depending on the group: a regular chow diet, consisting of protein 20% kcal, Fat 10% kcal, and Carbohydrate 70% kcal, energy density 3.82 kcal/g (rodent diet with 10 kcal % fat, D12450B, Research diets, New Brunswick, NJ); or an HFD, consisting of protein 20% kcal, Fat 60% kcal, and Carbohydrate 20% kcal, energy density 5.21 kcal/g (rodent diet with 60 kcal % fat, D12492, Research diets). Mice were intraperitoneally administered treatments from 6 to 42 weeks of age according to which group they were enrolled in, as follows: CN, chow diet and non-treatment control (saline, 200 μl/day); CP, chow diet and methylprednisolone treatment (5 mg/kg/day); HN, HFD and non-treatment control (saline, 200 μl/day); and HP, HFD and methylprednisolone treatment (5 mg/kg/day). The exact formulations of macronutrients and micronutrients for the chow and HFDs are presented in Supplementary Table 1. Mice were housed in an animal care facility at the CHA University in the CHA biocomplex in a temperature-controlled environment under a 12-h light/dark with access to food and water ad libitum. This study was reviewed and approved by the Institutional Animal Care and Use Committee of the CHA University (CE2018068). All procedures were performed in compliance with the Animal Welfare Act Regulations and Guide for the Care and Use of Laboratory Animals.

### Determination of proteinuria, anti-dsDNA antibody levels and blood urea nitrogen (BUN)

Blood samples were collected from mice under isoflurane anesthesia at 24, 32, and 40 weeks of age, and at autopsy, and the obtained serum samples were maintained at − 70 °C until the analysis. Anti-dsDNA antibody levels were measured using a mouse anti-dsDNA ELISA kit (Shibayagi Co., Ltd., Ishihara, Shibukawa, Japan)^54^. BUN levels were measured using urea nitrogen colorimetric detection kits (EIABUN; Invitrogen, Carlsbad, CA, USA).

### IPGTT, dual-energy X-ray absorptiometry (DEXA), and indirect calorimetry

These assays are described in detail in the supplementary information.

### Flow cytometry

These experiments are described in detail in the supplementary information^4^.

### Hematoxylin and eosin (H&E), periodic acid/Schiff (PAS) reagent, and Masson trichrome staining, and immunofluorescence analysis of kidneys

Mice were anesthetized with isoflurane and were sacrificed by cervical dislocation for harvesting at the end of the study (42–43 weeks of age). H&E, PAS, Masson trichrome staining, and immunofluorescence analysis of the kidneys were conducted as described in our previous study^4^. Mesangial proliferation, inflammatory cell infiltration, tubular dilation, and fibrosis were scored on a 4-point scale as follows: 0 (none), 1+ (mild), 2+ (moderate), 3+ (moderate to severe), and 4+ (severe). Fluorescence staining intensities of IgG or C3 deposits were graded as 0 (none), 1+ (mild), 2+ (moderate), 3+ (moderate to strong) and 4+ (strong).

### Determination of serum cytokine levels

Serum samples were assayed using Milliplex^®^ MAP Kits (Millipore, Burlington, Massachusetts, USA) for interferon-γ (IFN-γ), interleukin-1β (IL-1β), IL-2, IL-4, IL-6, IL-10, IL-12(p70), IL-15, IL-17a, TNF-α, chemokine (C-C motif) ligand 2 (CCL2 (MCP-1)), and IFN-inducible protein 10 (IP-10 (CXCL10)).

### Determination of serum adipokine levels

Serum samples from all mice were assayed using a multiplex adipokine kit for insulin, leptin, resistin, and plasminogen activator inhibitor-1 (PAI-1) (MADCYMAG-71K, Millipore, Bedford, MA, USA). Serum samples were further assayed for adiponectin using a Mouse Adiponectin/Acrp30 Immunoassay (R&D Systems, Minneapolis, MN, USA).

### Determination of serum levels of free fatty acids, glycerol, total cholesterol, and triglycerides

Serum levels of free fatty acids, glycerol, total cholesterol, and triglycerides were measured using free fatty acid quantification kits (ab65341, Abcam, Cambridge, UK), a glycerol colorimetric assay kit (10010755, Cayman Chemical, Ann Arbor, MI, USA), a total cholesterol colorimetric assay kit (MBS2540484, Mybiosource, San Diego, USA), and a triglyceride colorimetric assay kit (10010303, Cayman Chemical), respectively.

### Determination of cytokine levels in kidney extracts

Cytokine levels in the kidney extracts were determined as described in our previous study^4^. Briefly, the total protein concentrations of the kidney extracts were adjusted to 2 mg/ml and used for the analysis of IFN-γ, IL-1β, IL-4, IL-6, IL-10, IP-10, MCP-1, CCL4, and TNF-α using a Milliplex^®^ MAP Kit (Millipore).

### Statistical analysis

Except for proteinuria and the survival rate data, all results are expressed as mean ± SEM. Proteinuria and survival rate data were analyzed using Kaplan–Meier curves and the log-rank test. Urine protein/creatinine ratio (UP/C), anti-dsDNA antibody levels, BUN, IPGTT, and cytokine levels were compared using the Kruskal–Wallis test followed by the Mann–Whitney *U* test, while rest of the data were compared among groups using one-way ANOVA, followed by post-hoc Tukey's multiple-comparison tests. Statistical significance was set at *P* < 0.05. All statistical analyses were performed using SPSS version 24.0 (IBM, Armonk, NY, USA).

### Ethical statement

The study is reported in accordance with the ARRIVE guidelines.

### Ethics approval

This study was reviewed and approved by the Institutional Animal Care and Use Committee of the CHA University (CE2018068).

## Supplementary Information

Supplementary Information.

## Data Availability

The datasets used and/or analyzed during the current study are available from the corresponding author on reasonable request.
