# Supplementary material for: Effects of high fat diet-induced obesity on pathophysiology, immune cells, and therapeutic efficacy in systemic lupus erythematosus
Source: Sci Rep. 2022 Nov 2;12:18532. doi: 10.1038/s41598-022-21381-3 (PMC9630451; doi:10.1038/s41598-022-21381-3)
Supplement: Supplementary file 1 — Supplementary Information. [file 41598_2022_21381_MOESM1_ESM.pdf]

Supplementary Information

Title: Effects of high fat diet-induced obesity on pathophysiology, immune cells, and therapeutic efficacy in systemic lupus erythematosus

Eun Wha Choi[1*], Hee Je Kim[1], Yun Chan Jung[2], Hye Sun Go[3], Je Kyung Seong[3,4]

[1]Department of Veterinary Clinical Pathology, College of Veterinary Medicine & Institute of Veterinary Science, Kangwon National University, 1 Kangwondaehak-gil, Chuncheon, Gangwon-do, 24341, Republic of Korea

[2]Chaon, 331 Pangyoyeok-ro, Bundang-gu, Seongnam, Republic of Korea

[3]Laboratory of Developmental Biology and Genomics, BK21 Plus Program for Advanced Veterinary Science, Research Institute for Veterinary Science, College of Veterinary Medicine, and Korea Mouse Phenotyping Center, Seoul National University, 599 Gwanak-ro, Gwanak-gu, Seoul, 08826, Republic of Korea

[4]Interdiscplinary Program for Bioinformatics, Seoul National University, 599 Gwanak-ro, Gwanak-gu, Seoul, 08826, Republic of Korea

*Corresponding author: Eun Wha Choi, DVM, PhD, Assistant Professor

Address: Department of Veterinary Clinical Pathology, College of Veterinary Medicine, Kangwon National University, 1 Kangwondaehak-gil, Chuncheon-si, Gangwon-do 24341, Republic of Korea

Telephone: 82-33-250-8794, Fax: 82-33-259-5625

E-mail: ewchoi@kangwon.ac.kr

Materials and methods

Determination of proteinuria

During the experiments, urine protein levels were measured every two weeks. Fresh urine was collected via abdominal massage. Urine protein was measured using the Coomassie Brilliant Blue method as described in our previous study [54]. Urine creatinine at 42 weeks of age was measured using a creatinine assay (KGE005, R&D Systems, Minneapolis, MN, USA) with urine diluted in deionized water (1:20 dilution).

Intraperitoneal glucose tolerance test (IPGTT), dual-energy X-ray absorptiometry (DEXA), and indirect calorimetry

In the first experimental group, IPGTT was conducted at 36 weeks of age (all mice), and body fat percentage was measured using DEXA at 42 weeks of age (n = 6 per group). Food intake, activity, rearing, and energy expenditure were measured in six mice per group using indirect calorimetry at 42 weeks of age. For the IPGTT, mice were fasted overnight (18 h), and glucose (2 g/kg body weight) was injected intraperitoneally. Blood samples were taken at 0, 15, 30, 60, 90, and 120 min for blood glucose analyses using a glucometer (Accu-Chek).

Flow cytometry

Single-cell suspensions were obtained from the spleens of NZB/W F1 mice at autopsy (42-43 weeks of age). An Fc blocking antibody was used to prevent non-specific binding (anti-mouse CD16/32, BioLegend, San Diego, CA, USA). The splenocytes were stained with peridinin

chlorophyll protein complex-conjugated anti-mouse CD45 (PerCP-CD45, 1.25 μl/well, BioLegend), allophycocyanin-conjugated anti-mouse CD3e (APC-CD3e, 1 μl/well, eBioscience, San Diego, CA, USA), fluorescein isothiocyanate (FITC)-conjugated anti-mouse CD4 (FITC-CD4, 2 μl/well, BD Biosciences, San Jose, CA, USA), and PE-cyanine7-conjugated anti-mouse CD8a (0.5 μl/well, eBioscience).

T cell profiles were analyzed as described previously [4]; briefly, we examined the proportions of Th1 cells (CD4+CD25+T-bet+), Th2 cells (CD4+CD25+GATA-3+), Th17 cells (CD4+CD25+ROR-γt+), and Treg cells (CD4+CD25+Foxp3+). To analyze T helper subsets, splenocytes were stained with antibodies against CD4 and CD25 (FITC-conjugated anti-mouse CD4, 2 μl/well, and APC-conjugated anti-mouse CD25, 1 μl/well, BD Biosciences). Cells were fixed and permeabilized prior to staining with T-bet, GATA-3, Foxp3, and ROR-γt antibodies (PE-, 2 μl/well, 4 μl/well, 0.5 μl/well, and 2 μl/well, respectively, BD Biosciences).

Macrophage subset was analyzed; briefly, we examined proportion of M1 (CD45+ CD64+ CD11c+ CD206-) and M2 (CD45+ CD64+ CD11c- CD206+) cells using PerCP-conjugated anti-mouse CD45 (1.25 μl/well, BioLegend), APC-conjugated anti-mouse CD64 (5 μl/well, BioLegend), PE-conjugated anti-mouse CD11c (1.25 μl/well, BioLegend), and Alexa Fluor 488-conjugated anti-mouse CD206 (2 μl/well, BioLegend).

To obtain the stromal vascular fraction (SVF) from mouse epididymal white adipose tissue (eWAT), white fat was isolated from the adipose tissue after being placed in 10 mL of fresh cold PBS. Adipose tissue was gently chopped into 1–2 mm pieces and mixed with collagenase II solution (400 U/mL). The mixture was incubated at 37°C for 20 min with gentle shaking. After isolation, SVF was separated from adipocytes by centrifugation at 470 ×g for 5 min. An Fc blocking antibody was used to prevent non-specific binding (anti-mouse CD16/32,

BioLegend). Next, the SVF cells were incubated with antibodies for surface staining at 4°C for 30 min. The cells were washed with PBS containing 2% fetal bovine serum. FASCAria II (BD Biosciences) and BD FACSDiva software (BD Biosciences) were used for the flow cytometry analysis.

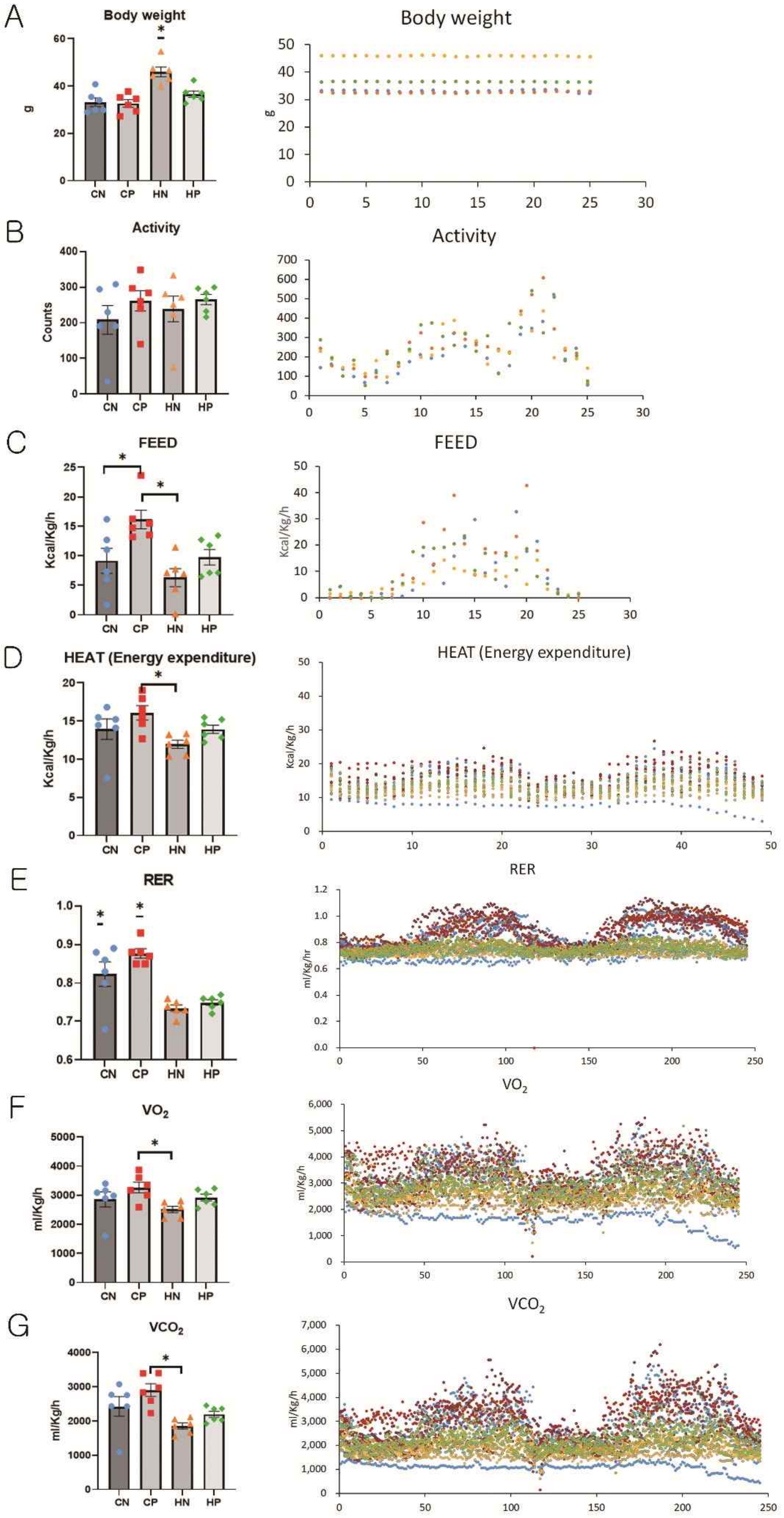
A
Body weight
g
CN CP HN HP
Body weight
g
B
Activity
Counts
CN CP HN HP
Activity
C
FEED
Kcal/Kg/h
CN CP HN HP
FEED
Kcal/Kg/h
D
HEAT (Energy expenditure)
Kcal/Kg/h
CN CP HN HP
HEAT (Energy expenditure)
Kcal/Kg/h
E
RER
CN CP HN HP
RER
ml/Kg/hr
F
VO2
ml/Kg/h
CN CP HN HP
VO2
ml/Kg/h
G
VCO2
ml/Kg/h
CN CP HN HP
VCO2
ml/Kg/h

Supplementary Figure 1. Indirect calorimetry. (A) Body weight, (B) Activity, (C) food uptake, (D) Energy expenditure (heat), (E) Respiratory energy ratio, (F) Oxygen consumption (VO_2), and (G) Carbon dioxide production (VCO_2). Data were compared among groups using one-way analysis of variance (ANOVA) followed by post hoc Tukey's multiple-comparison tests. A *P*-value of < 0.05 was considered statistically significant (n = 6 per group). CN: chow diet and non-treatment control (saline, 200 μl/day), CP: chow diet and methylprednisolone treatment (5 mg/kg/day), HN: high fat-diet and non-treatment control (saline, 200 μl/day), and HP: high fat-diet and methylprednisolone treatment (5 mg/kg/day).

Supplementary Table 1. Formulation of the chow and high-fat diets.

Chow diet (Research diets D12450B, Rodent Diet with 10 kcal% Fat)

Formulation

Class description	Ingredients	Grams
Protein	Casein, Lactic, 30 Mesh	200.00 g
Protein	Cystine, L	3.00 g
Carbohydrate	Sucrose, Fine Granulated	354.00 g
Carbohydrate	Starch, Corn	315.00 g
Carbohydrate	Lodex 10	35.00 g
Fiber	Solka Floc, FCC200	50.00 g
Fat	Soybean Oil, USP	25.00 g
Fat	Lard	20.00 g
Mineral	S10026B	50.00 g
Vitamin	Choline Bitartrate	2.00 g
Vitamin	V10001C	1.00 g
Dye	Dye, Yellow FD&C #5, Alum. Lake 35-42%	0.05 g
	Total:	1055.05 g

High-fat diet (Research diets D12492, Rodent Diet with 60 kcal% Fat)

Formulation

Class description	Ingredients	Grams
Protein	Casein, Lactic, 30 Mesh	200.00 g
Protein	Cystine, L	3.00 g
Carbohydrate	Lodex 10	125.00 g
Carbohydrate	Sucrose, Fine Granulated	72.80 g
Fiber	Solka Floc, FCC200	50.00 g
Fat	Lard	245.00 g
Fat	Soybean Oil, USP	25.00 g
Mineral	S10026B	50.00 g
Vitamin	Choline Bitartrate	2.00 g
Vitamin	V10001C	1.00 g
Dye	Dye, Blue FD&C #1, Alum. Lake 35-42%	0.05 g
	Total:	773.85 g
